# Quadrivalent Human Papillomavirus (HPV) Vaccine Induces HPV-Specific Antibodies in the Oral Cavity: Results From the Mid-Adult Male Vaccine Trial

**DOI:** 10.1093/infdis/jiw359

**Published:** 2016-08-10

**Authors:** Ligia A. Pinto, Troy J. Kemp, B. Nelson Torres, Kimberly Isaacs-Soriano, Donna Ingles, Martha Abrahamsen, Yuanji Pan, Eduardo Lazcano-Ponce, Jorge Salmeron, Anna R. Giuliano

**Affiliations:** 1 HPV Immunology Laboratory, Leidos Biomedical Research, Inc., Frederick National Laboratory for Cancer Research, Maryland; 2 Center for Infection Research in Cancer, Moffitt Cancer Center, Tampa, Florida; 3 National Institute of Public Health, Cuernavaca, Mexico

**Keywords:** HPV vaccine, males, serum, mouthwash, saliva

## Abstract

***Background.*** Human papillomavirus virus type 16 (HPV-16) and HPV-18 cause a large proportion of oropharyngeal cancers, which are increasing in incidence among males, and vaccine efficacy against oral HPV infections in men has not been previously evaluated.

***Methods.*** Sera and saliva collected in mouthwash and Merocel sponges at day 1 and month 7 were obtained from 150 men aged 27–45 years from Tampa, Florida, and Cuernavaca, Mexico, who received Gardasil at day 1 and months 2 and 6. Specimens were tested for anti–HPV-16 and anti–HPV-18 immunoglobulin G (IgG) levels by an L1 virus-like particle–based enzyme-linked immunosorbent assay.

***Results.*** All participants developed detectable serum anti–HPV-16 and anti–HPV-18 antibodies, and most had detectable antibodies in both oral specimen types at month 7 (HPV-16 was detected in 93.2% of mouthwash specimens and 95.7% of sponge specimens; HPV-18 was detected in 72.1% and 65.5%, respectively). Antibody concentrations in saliva were approximately 3 logs lower than in serum. HPV-16– and HPV-18–specific antibody levels, normalized to total IgG levels, in both oral specimen types at month 7 were significantly correlated with serum levels (for HPV-16, ρ was 0.90 for mouthwash specimens and 0.92 for sponge specimens; for HPV-18, ρ was 0.89 and 0.86, respectively).

***Conclusions.*** This is the first study demonstrating that vaccination of males with Gardasil induces HPV antibody levels at the oral cavity that correlate with circulating levels.

Human papillomaviruses (HPVs) cause approximately 70% of oropharyngeal cancers (OPCs), with the majority due to HPV-16 [1, 2]. US incidence rates of HPV-positive OPCs are increasing, with a concomitant increase in the proportion of OPCs attributable to HPV [1]. The incidence of OPC is significantly higher among males, compared with females [3], and to date, there is not a routine screening method available to detect HPV-related OPCs at early stages.

The quadrivalent HPV vaccine (Gardasil) is highly efficacious in reducing anogenital HPV-6, -11, -16, and -18 infections, subsequent development of HPV-related external genital lesions, and anal diseases in young males (ages 16–26 years) [4]. These findings led to the licensure of Gardasil for use in males ages 9–26 years for the prevention of HPV-6– and HPV-11–related genital warts and HPV-16– and HPV-18–related anal cancers. Recently, it has been demonstrated that mid-adult-aged men, who are more likely than males of other ages to develop HPV-related cancer, develop a strong antibody response to Gardasil [5]. Successful prevention of HPV-related cancers depends on local, long-term protection against acquisition of infection. Currently licensed HPV L1 vaccines induce strong systemic antibody responses to the HPV types that are included in these vaccines, and antibodies are believed to be responsible for providing the high efficacy against HPV infection observed in vaccinated individuals.

Post hoc vaccine trial evidence suggests that the bivalent HPV vaccine protects against 1-time-detected prevalent oral infections in females [6]. However, this trial did not determine whether HPV immunization resulted in HPV-specific antibody levels at the oral cavity and did not investigate the determinants of oral antibody levels. Most immunogenicity studies have been conducted in serum and not at the relevant sites of infection, owing to the complexities of testing at mucosal sites. Thus, the levels of antibodies present at the oral cavity following vaccination and the levels required for protection against oral HPV infection are unknown. This information is important to determine the potential to prevent HPV-related oral cancers in men with a vaccine approach and to design a phase 3 clinical trial in mid-adult-aged males to evaluate the efficacy of HPV vaccines to reduce OPC incidence.

Hence, we assessed the levels of HPV-16– and HPV-18–specific immunoglobulin G (IgG) antibodies at the oral cavity following a phase 2 vaccine trial of mid-adult-aged men (ages 27–45) from the United States and Mexico who received 3 doses of Gardasil [5]. We also determined the associations between oral and serum HPV-16 and HPV-18 IgG antibody levels, as well as factors associated with oral antibody levels induced by vaccination. This is the first study of its kind evaluating oral mucosa HPV immunity in males following HPV vaccination.

## METHODS

### Study Population

The Mid-Adult Men study (clinical trials registration NCT01432574) is a single-arm intervention trial that enrolled, vaccinated, and assessed the circulating antibody response to Gardasil in men aged 27–45 years [5]. Men were solely selected for this study because of the 3–4-fold higher incidence of OPC among males as compared to females [3]. Briefly, subjects were vaccinated intramuscularly with Gardasil at day 1 of the study and at months 2 and 6. A total of 150 men from Tampa, Florida, and Cuernavaca, Mexico, who met eligibility criteria (male sex, age 27–45 years, and completion of 4 years of follow-up in the HPV Infection in Men study) were enrolled and received at least 1 dose of vaccine. A subset of vaccine recipients had mouthwash gargle (n = 147), Merocel sponge (n = 139), and serum (*n* = 126) specimens available for HPV antibody testing both at day 1 and month 7. Computerized questionnaires were completed by subjects to collect demographic, behavioral, and oral health–related (ie, presence of gum disease, tooth loss, gum bleeding, and mouth warts) data. Race was collected in the questionnaire as a self-reported assessment, and this demographic question contained several options to select from. Each participant underwent a clinical examination on day 1 and at month 7 (1 month after dose 3) and completed questionnaires at both visits to record sexual behavior and other risk factors for HPV infection. At the day 1 and month 7 study visits, blood and oral specimens (mouthwash gargle and Merocel sponge) were collected. The institutional review boards at each participating center (University of South Florida in the United States and Instituto Nacional de Salud Publica in Mexico) approved the protocol, and informed consent was obtained from all subjects. The study was conducted in conformance with applicable country or local requirements regarding ethical committee review, informed consent, and other statutes or regulations regarding the protection of the rights and welfare of human subjects participating in biomedical research.

### Specimens

Ten-milliliter blood specimens were collected in a red-top tube. Following centrifugation, sera were aliquoted into cryovials and stored at −80°C until testing. Oral fluid from each participant was collected in both Merocel sponges and in mouthwash gargles. Subjects first placed a Merocel sponge against the central part of the inner cheek for 15 seconds, without touching other parts of the mouth (ie, teeth and tongue) [7]. The participant then flipped the sponge to allow the other side of the sponge to come into contact with the mucosa for 15 seconds. After 30 seconds, the sponge was placed into a sterile 10 mL cryovial. A single lot of Merocel sponges was used per site. To reduce sponge weight variability, unused sponges of the same lot were used to calculate mean sponge weight. Vials were stored at −80*°*C prior to extraction and testing. Mouthwash gargle specimens were collected by use of a 30-second rinse/gargle with 15 mL of mouthwash solution [8]. Supernatants were stored at −80°C prior to antibody testing. These collection methods and validated assays have been used in a number of clinical epidemiologic studies [7–9].

Oral fluid was extracted from Merocel sponges according to a previously published protocol [7]. A total of 300 µL of extraction buffer (phosphate-buffered saline; Invitrogen, Grand Island, New York; 1.5% NaCl, Sigma-Aldrich, St. Louis, Missouri; and 100 μg/mL aprotinin, Sigma-Aldrich) was added to the top of each sponge. The sponges were incubated at 4°C for 30 minutes prior to centrifugation at 13 000 × *g* for 15 minutes at 4°C. An additional 300 μL of extraction buffer was added to each sponge and immediately centrifuged. Prior to adding 4 μL of fetal calf serum for storage, 20 μL of extract was saved for protein analysis (Pierce BCA, Thermo Scientific, Waltham, Massachusetts). To account for variations in the amount of oral fluid collected between participants, the antibody levels measured in oral fluid extracted from the sponges were normalized on the basis of weight, using the following formula: [specimen weight (in grams) − mean dry sponge weight (in grams) + 0.6 g]/[specimen weight (in grams) − mean dry sponge weight (in grams)]; “0.6 g” refers to the weight of the extraction buffer added to each specimen.

### Direct L1 Virus-Like Particle (VLP)–Based Enzyme-Linked Immunosorbent Assay (ELISA)

Anti-HPV IgG antibodies were detected by an ELISA, as previously described [10–12]. This ELISA measures total levels of HPV-16– and HPV-18–specific IgG antibodies (both neutralizing and nonneutralizing) and is amenable for use in large epidemiologic and clinical studies. The assay is highly reproducible, with a reported overall coefficient of variation of 11.4% [10]. Briefly, polystyrene flat-bottomed microtiter plates (MaxiSorp, high binding; Nunc, Thermo Fisher Scientific) were coated with HPV-16 or HPV-18 L1 VLPs and incubated at 4°C. Prior to use, the plates were washed with a phosphate-buffered saline containing 0.25% Tween 20. After blocking the plates with blocking buffer containing 4% skim milk and 0.2% Tween 20 in phosphate-buffered saline, the plates were washed again. Serum (starting dilution 1:100) and oral fluids (mouthwash gargle and Merocel sponge; starting dilution, 1:2) from participants were serially diluted in the blocking solution in 2-fold increments in the assay plate. The plates were incubated for 1 hour at room temperature. After plates were washed 4 times, a solution of peroxidase-labeled goat anti-human IgG (KPL, Gaithersburg, Maryland) was added for 1 hour at room temperature. Plates were then developed with a tetramethylbenzidine substrate solution (KPL) for 25 minutes in the dark at room temperature. Next, the reaction was stopped, and the absorbance was measured with a microtiter plate reader (Spectramax M5; Molecular Devices, Sunnyvale, California). Antibody levels, expressed as ELISA units (EU) per milliliter, were calculated by interpolation of ODs from the standard curve by averaging the calculated concentrations from all dilutions that fall within the working range of the standard curve. The seropositivity lower cut points for serum were set at 19 EU/mL for anti–HPV-16 and 18 EU/mL for anti-HPV-18 [13]. Cut points for mouthwash gargles were set at 0.042 EU/mL for anti–HPV-16 and 0.032 EU/mL for anti–HPV-18, and cut points for Merocel sponges were set at 0.030 EU/mL for anti–HPV-16 and 0.036 EU/mL for anti–HPV-18. Samples with levels lower than the cut points were given a value of one half of the cut point value.

### Total IgG ELISA

Total human IgG levels were measured in duplicate in each specimen type (serum, mouthwash gargle, and Merocel sponge), using an ELISA according to the manufacturer's protocol (Bethyl Laboratories, Montgomery, Texas) [7]. Total IgG levels in each different sample type (serum, mouthwash gargle, and Merocel sponge) were used to normalize levels of HPV-specific antibodies across different biological specimens and to compare levels between different collection time points.

### Statistical Analysis

We conducted an intent-to-treat analysis that included all men in the trial regardless of HPV DNA and antibody status at enrollment. The *P* values from the demographic parameters were calculated using Pearson χ^2^ analysis (Monte Carlo), for categorical variables, and the Wilcoxon rank sum test (Monte Carlo), for continuous variables. The proportions of men who had measurable serum or oral HPV-16/18 IgG, as well as associated 95% confidence intervals (CIs), were estimated. Geometric mean titers (GMTs) and 95% CIs for HPV-16 and HPV-18 antibody levels were calculated and reported by categories of the covariates examined. GMTs were compared across groups, using the Wilcoxon rank sum test or the Kruskal–Wallis test. Correlations between serum-specific and oral-specific IgG levels were determined by Spearman correlation coefficients. Among trial participants with detectable antibodies in both serum and oral fluid (mouthwash gargle and Merocel sponge), HPV-specific antibody levels were normalized to the total IgG level in the serum or oral specimen, respectively, and are reported as ratios of the HPV-specific IgG concentrations to the total concentrations of IgG. In Supplementary Figure 1, *P* values were calculated by the Wilcoxon rank sum test, and Bonferroni correction was used to set significance at a *P* value of < .0167.

## RESULTS

A total of 150 men were enrolled in this study, but only a subset of men with specimens available at both baseline (day 1) and month 7 (1 month after the third dose of vaccine) visits were tested and included in this analysis (147 mouthwash gargle, 139 Merocel sponge, and 126 serum specimens). Table 1 shows the most relevant demographic features for oral mucosa measurements for this cohort. The median age of the participants was 36 years (range, 27–45 years). Significant differences were observed by country for smoking status, gum bleeding, frequency of tooth brushing, and HPV-18 serostatus at day 1. Overall, very few men were positive for oral HPV-16 DNA (2.7%) or HPV-18 DNA (0.7%) at day 1.

**Table 1.** Demographic Characteristics of the Study Participants at Baseline

Characteristic	Subjects, No.	Overall^a^	Subjects, No.	Subjects in the US^a^	Subjects, No.	Subject in Mexico^a^	*P* Value
Age at visit, y							
Mean ± SD	150	34.8 ± 5.1	75	34.7 ± 5.5	75	35 ± 4.7	.67
Median (range)	150	36 (27–45)	75	35 (27–45)	75	36 (27–45)	
Race							
White	…	68 (45.3)	…	58 (77.3)	…	10 (13.3)	
Black/African American	…	12 (8)	…	12 (16)	…	0 (0)	<.001
Other	…	70 (46.7)	…	5 (6.7)	…	65 (86.7)	
Any alcohol intake							
Yes	…	112 (77.2)	…	59 (78.7)	…	53 (75.7)	.69
No	…	33 (22.8)	…	16 (21.3)	…	17 (24.3)	
Monthly alcohol intake							
Mean ± SD	112	38.6 ± 70.3	59	42.8 ± 77.5	53	33.9 ± 61.8	.07
Median (range)	112	18 (1–576)	59	24 (2–576)	53	12 (1–396)	
Smoking status							
Current	…	37 (25.5)	…	9 (12)	…	28 (40)	
Former	…	24 (16.6)	…	16 (21.3)	…	8 (11.4)	<.001
Never	…	84 (57.9)	…	50 (66.7)	…	34 (48.6)	
Gum disease							
Yes	…	28 (19.3)	…	16 (21.3)	…	12 (17.1)	.55
No	…	117 (80.7)	…	59 (78.7)	…	58 (82.9)	
Tooth loss							
0 teeth	…	131 (93.6)	…	72 (96)	…	59 (90.8)	.30
≥1 tooth	…	9 (6.4)	…	3 (4)	…	6 (9.2)	
Gum bleeding							
Yes	…	26 (17.9)	…	5 (6.7)	…	21 (30)	<.001
No	…	119 (82.1)	…	70 (93.3)	…	49 (70)	
Tooth brushing, episodes, daily no.							
Mean ± SD	145	2 ± 0.7	75	1.7 ± 0.6	70	2.4 ± 0.7	<.001
Median (range)	145	2 (1–4)	75	2 (1–3)	70	2 (1–4)	
Mouth warts							
Yes	…	3 (2.1)	…	3 (4)	…	0 (0)	.26
No	…	142 (97.9)	…	72 (96)	…	70 (100)	
Oral sex frequency, episodes, no.							
Mean ± SD	98	16.1 ± 23.5	50	19.8 ± 24.1	48	12.3 ± 22.4	.06
Median (range)	98	6 (1–150)	50	10 (1–100)	48	6 (1–150)	
Oral HPV-16 DNA status							
Negative	…	146 (97.3)	…	74 (98.7)	…	72 (96)	.62
Positive	…	4 (2.7)	…	1 (1.3)	…	3 (4)	
Oral HPV-18 DNA status							
Negative	…	149 (99.3)	…	75 (100)	…	74 (98.7)	1.00
Positive	…	1 (0.7)	…	0 (0)	…	1 (1.3)	
Genital HPV-16 DNA status							
Negative	…	134 (89.3)	…	66 (88)	…	68 (90.7)	.79
Positive	…	16 (10.7)	…	9 (12)	…	7 (9.3)	
Negative genital HPV-18 DNA status	…	150 (100)	…	75 (100)	…	75 (100)	NE
HPV-16 serostatus (day 1)							
Negative	…	102 (81)	…	44 (86.3)	…	58 (77.3)	.26
Positive	…	24 (19)	…	7 (13.7)	…	17 (22.7)	
HPV-18 serostatus (day 1)							
Negative	…	100 (79.4)	…	46 (90.2)	…	54 (72)	.01
Positive	…	26 (20.6)	…	5 (9.8)	…	21 (28)	

Abbreviations: HPV, human papillomavirus; NE, not estimable; SD, standard deviation.

^a^ Unless otherwise indicated, data are number (percentage) of study participants.

The level of HPV-16 and HPV-18 antibodies induced by vaccination in serum and oral fluid collected in gargles and in sponges are shown in Table 2. At day 1, 19%–21% of the total population had detectable serum anti–HPV-16 or anti–HPV-18 antibodies; however, levels among the positive individuals were low (HPV-16, 34.46 EU/mL; HPV-18, 26.89 EU/mL). As previously reported using the chemiluminescence immunoassay [5], all men developed strong antibody responses to HPV-16 and HPV-18 in serum following vaccination, regardless of the country of residence. Serum anti–HPV-16 and anti–HPV-18 GMTs were 2119.8 and 611.6 EU/mL, respectively, at month 7. Anti–HPV-16 antibody levels correlated with HPV-18 antibody levels (ρ = 0.66; *P* < .001).

**Table 2.** Individuals With Detectable Anti–Human Papillomavirus Virus Type 16 (HPV-16) and HPV-18 Antibodies Before and After Vaccination in Oral Fluids and Serum

HPV Type, Specimen Type, Clinic	Samples, No.		Day 1	Month 7	
		Ab Positive,^a^ No. (%)	GMT^b^ (95% CI)	Ab Positive,^a^ No. (%)	GMT^b^ (95% CI)
16					
Sponge					
US	64	8 (12.5)	0.44 (.21, .92)	59 (92.2)	2.49 (1.72, 3.58)
Mexico	75	0 (0.0)	…	74 (98.7)	6.60 (5.25, 8.28)
Total	139	8 (5.8)	0.44 (.21, .92)	133 (95.7)	4.28 (3.44, 5.33)
Mouthwash					
US	72	0 (0.0)	…	62 (86.1)	0.17 (.14, .20)
Mexico	75	0 (0.0)	…	75 (100.0)	0.33 (.27, .41)
Total	147	0 (0.0)	…	137 (93.2)	0.24 (.21, .28)
Serum					
US	51	7 (13.7)	35.40 (19.66, 63.71)	51 (100.0)	2078.34 (1640.51, 2633.02)
Mexico	75	17 (22.7)	34.09 (27.68, 41.98)	75 (100.0)	2148.40 (1836.81, 2512.84)
Total	126	24 (19.0)	34.46 (28.27, 42.01)	126 (100.0)	2119.76 (1858.22, 2418.11)
18					
Sponge					
US	64	10 (15.6)	0.66 (.34, 1.28)	36 (56.3)	1.39 (.95, 2.04)
Mexico	75	2 (2.7)	5.45 (.00, 66 576.25)	55 (73.3)	3.26 (2.50, 4.24)
Total	139	12 (8.6)	0.93 (.43, 2.03)	91 (65.5)	2.33 (1.84, 2.93)
Mouthwash					
US	72	1 (1.4)	…	45 (62.5)	0.08 (.07, .10)
Mexico	75	5 (6.7)	0.07 (.03, .13)	61 (81.3)	0.15 (.12, .18)
Total	147	6 (4.1)	0.07 (.04, .12)	106 (72.1)	0.12 (.10, .13)
Serum					
US	51	5 (9.8)	27.21 (20.11, 36.81)	51 (100.0)	603.74 (466.19, 781.86)
Mexico	75	21 (28.0)	26.81 (22.88, 31.41)	75 (100.0)	617.02 (512.43, 742.96)
Total	126	26 (20.6)	26.89 (23.57, 30.67)	126 (100.0)	611.61 (526.41, 710.6)

Abbreviation: CI, confidence interval.

^a^ Defined as an antibody (Ab) response above the cutoff.

^b^ Defined as the geometric mean titer (GMT) among positive values (ie, values above the cutoff).

At day 1, <9% of the participants had detectable anti–HPV-16 or anti–HPV-18 levels in any of the 2 oral fluid specimens tested. Following vaccination, most participants had measurable HPV-16– and HPV-18–specific antibodies in oral fluids (HPV-16 was detected in 93.2% of mouthwash gargle specimens and 95.7% of Merocel sponge specimens; HPV-18 was detected in 72.1% of mouthwash gargle specimens and 65.5% of Merocel sponge specimens; Table 2). Detectability of anti–HPV-18 antibodies after vaccination was slightly higher in mouthwash gargle specimens than in Merocel sponge extracts, and, in general, levels of anti–HPV-18 antibodies were lower than levels of anti–HPV-16 antibodies for all specimen types.

**Figure 1.**
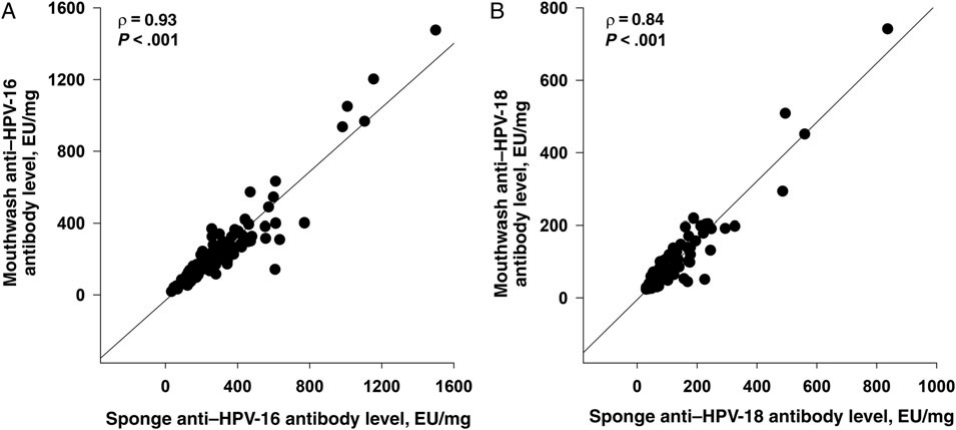
Correlations between human papillomavirus type 16 (HPV-16; *A*) and HPV-18 (*B*) antibody levels in mouthwash and sponges at month 7. Following receipt of 3 doses of Gardasil, the participants' samples at month 7 were tested for anti–HPV-16 and anti–HPV-18 antibodies and total immunoglobulin G (IgG) levels. Levels of antibodies determined by enzyme-linked immunosorbent assay (ELISA) were normalized to total IgG levels in the respective samples, and the values are reported as antibody titers in ELISA units (EU)/mL per mg of IgG. Spearman coefficient correlations were determined amongst samples with detectable levels of antibodies.

**Figure 2.**
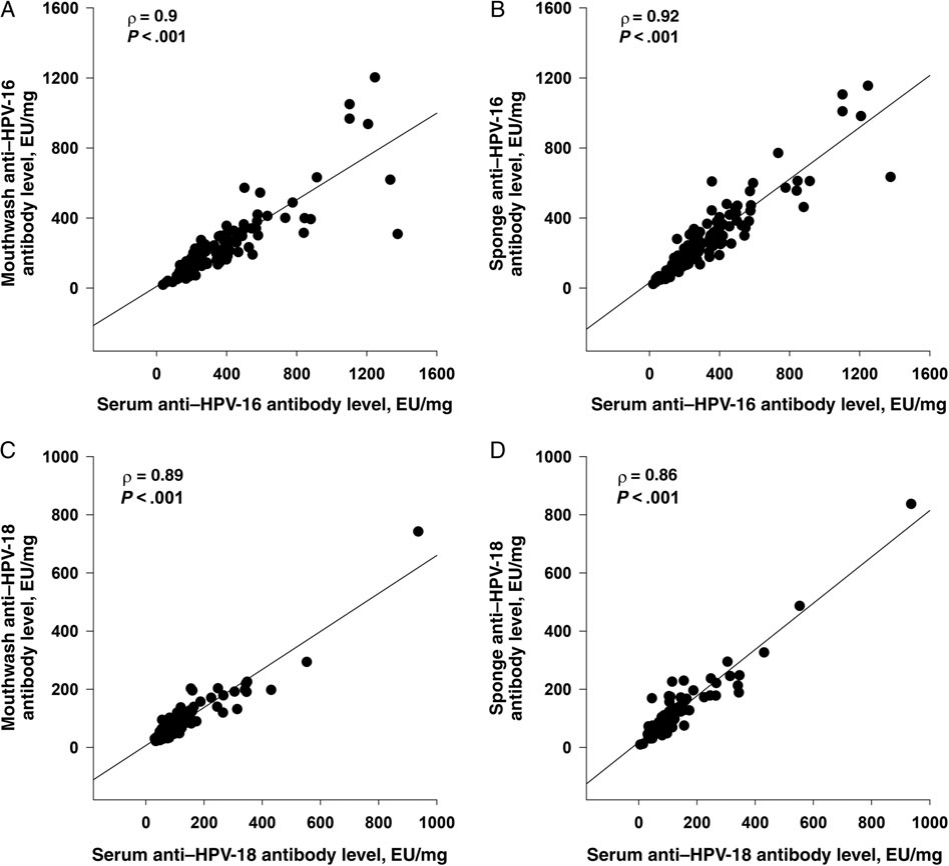
Correlations between human papillomavirus type 16 (HPV-16) and HPV-18 antibodies in oral fluids and serum at month 7. Following receipt of 3 doses of Gardasil, the participants' samples (sponge, mouthwash, and serum) were tested for anti–HPV-16 and anti–HPV-18 antibodies and total immunoglobulin G (IgG) levels. Levels of antibodies determined by enzyme-linked immunosorbent assay (ELISA) were normalized to total IgG levels in the respective samples, and the values are reported as antibody titers in ELISA units (EU)/mL per mg of IgG. Spearman coefficient correlations were determined amongst samples with detectable levels of antibodies.

HPV-specific antibody levels after vaccination in Merocel sponge specimens were approximately 262–495-fold lower than in serum (HPV-16, 4.28 vs 2119.76 EU/mL; and HPV-18, 2.33 vs 611.61 EU/mL; Table 2). As the measurement of oral HPV-specific antibody levels is dependent on the amount of oral fluid collected, we adjusted for differences in the collected volume by normalizing antibody levels to the corresponding total sample IgG concentration. As shown in Supplementary Figure 1 and Table 3, anti–HPV-16–specific antibody levels in oral fluid specimens, normalized to IgG levels, were only 1.18–1.49-fold lower than corresponding levels in serum and were not affected by country of residence. Normalized anti–HPV-18 antibody levels in mouthwash gargle specimens were identical to those in serum specimens (73.67 vs 76.19 EU/mg; Table 3) and 1.34-fold lower in mouthwash specimens than in Merocel sponge specimens. Anti–HPV-16 and anti–HPV-18 concentrations, normalized to IgG levels, were 1.26-fold and 1.34-fold lower in mouthwash gargle specimens than concentrations found in Merocel sponge specimens (Supplementary Figure 1). Strong correlations were observed between the levels of anti–HPV-16 and anti–HPV-18 antibodies found in the 2 oral sample types (ρ = 0.93 and ρ = 0.84 for HPV-16 and HPV-18, respectively; Figure 1*A* and 1*B*). In addition, HPV-16– and HPV-18–specific antibody levels at month 7 in both oral fluid specimens were significantly correlated with serum levels (for HPV-16, ρ was 0.90 for mouthwash gargle specimens and 0.92 for Merocel sponge specimens; for HPV-18, ρ was 0.89 and 0.86, respectively; Figure 2*A*–*D*).

**Table 3.** Immunoglobulin G (IgG)–Normalized Human Papillomavirus Type 16 (HPV-16) and HPV-18 Antibody Levels in Sponges, Mouthwash, and Serum Specimens, by Country, at Month 7

Country	Sponge, GMT, EU/mg (95% CI)^a^				Mouthwash, GMT, EU/mg (95% CI)^a^				Serum, GMT, EU/mg (95% CI)^a^		
	Samples, No.^b^	Anti–HPV-16	Samples, No.^b^	Anti–HPV-18	Samples, No.^b^	Anti–HPV-16	Samples, No.^b^	Anti–HPV-18	Samples, No.^b^	Anti–HPV-16	Anti–HPV-18
US	59	226.97 (185.04, 278.40)	36	94.78 (70.74, 126.98)	62	192.17 (159.85, 231.01)	45	83.91 (68.06, 103.46)	51	251.92 (195.41, 324.77)	73.18 (55.72, 96.12)
Mexico	74	221.44 (188.81, 259.71)	55	101.23 (84.72, 120.97)	75	165.55 (139.34, 196.69)	61	66.93 (55.94, 80.07)	75	272.68 (233.09, 319.01)	78.31 (64.97, 94.41)
Total	133	223.87 (197.55, 253.71)	91	98.63 (84.49, 115.13)	137	177.11 (156.31, 200.67)	106	73.67 (64.32, 84.37)	126	264.08 (230.29, 302.83)	76.19 (65.29, 88.92)

^a^ Defined as the geometric mean titer (GMTs) among positive values (ie, values above the cutoff) and normalized to total IgG levels in the corresponding sample type.

^b^ Data are no. of samples with detectable antibody levels.

Serum anti–HPV-16 antibody levels were not influenced by any of the demographic factors examined (Supplementary Table 1). Serum HPV-18 antibody levels were inversely associated with alcohol intake but were not affected by any other demographic factors. HPV-16 antibody levels in oral fluids differed by country, tobacco use, and frequency of tooth brushing and gum bleeding. HPV-18 antibody levels in oral fluids differed by country in both specimen types and by gum bleeding in mouthwash gargles. These effects were no longer observed when antibody levels in oral fluids were normalized to total oral IgG levels (Supplementary Table 2).

## DISCUSSION

To date, no vaccine trials have evaluated quadrivalent HPV vaccine efficacy against persistent oral HPV infections, the presumed obligate precursor to OPC. Gardasil has demonstrated robust efficacy against HPV infection at the cervix, vulva, and vagina in females and at the external genital skin and anal canal in men [4, 14, 15]. High levels of serum HPV-specific antibodies against vaccine types are induced by HPV vaccination [13, 16]. In preclinical studies, HPV antibodies have been shown to mediate type-specific protection [16]. As such, HPV antibodies are believed to be the main effectors of protection and efficacy against HPV infection at various mucosal sites. However, until now levels of antibodies at the site of infection (in this case, at the oral cavity) and covariates of those local antibody levels have not been reported. It was also unknown whether antibody levels in oral fluid would reflect systemic levels and levels found at other mucosal sites where HPV causes cancer. These important gaps in the field motivated us to conduct the first study, to our knowledge, to evaluate local antibody responses at the oral cavity to an HPV vaccine in males. Here, we demonstrate that vaccination with Gardasil induces detectable levels of anti-HPV antibodies at the oral cavity and that oral antibody levels reflect levels elicited systemically. One hundred percent of men seroconverted, and the majority of individuals developed detectable anti–HPV-16 and anti–HPV-18 antibodies (up to 96% and 72%, respectively) at the oral cavity following vaccination.

Using 2 independent oral sampling methods, HPV-16– and HPV-18–specific antibodies were detectable at the oral cavity in the majority of HPV vaccine recipients. A strong correlation was observed between antibody levels measured in mouthwash gargle specimens and in Merocel sponge extracts. These findings suggest that both methods could be used to monitor oral antibody levels in HPV vaccine studies. For some studies, mouthwash gargle specimen collection will be more convenient, as this collection method is commonly used for HPV genotyping [9, 17, 18]. Nevertheless, there is a global need for standardization of oral collection procedures for evaluation of mucosal antibodies and other biomarkers of interest in HPV vaccine trials. Oral antibody levels were much lower (by approximately 500-fold) than levels found in serum, but when normalized to specimen-specific IgG levels, oral and serum antibody levels differed by <1.5-fold. These findings and the observation that antibody levels correlated well in serum and oral fluids, when normalized by the total IgG level, support the concept that the majority of HPV-specific antibodies detected in the oral mucosa are likely to be antibodies that transudate from the peripheral blood, as described in the context of other vaccines [19, 20]. Consistent with this is our observation of an association between unadjusted levels of HPV antibodies and gum bleeding and/or tooth brushing frequency. This may explain the higher unadjusted levels of HPV antibodies found in the oral fluid of Mexican participants, where gum bleeding was reported at a higher frequency than among US trial participants. Upon adjustment for total IgG level, those associations were no longer observed. Our observed levels of oral IgG are in agreement with levels reported in other studies [7, 21, 22]. Although HPV-specific responses have been detected in oral fluid in females following HPV vaccination [23], methods previously used were not standardized or normalized, precluding comparisons of HPV antibody levels between studies. In addition, the previous study among females did not address whether local levels correlated with serum levels. In this work, IgG-normalized HPV-16 and HPV-16 antibody levels were not associated with any of the demographic features evaluated, indicating that the proportion of HPV-16–specific antibodies within total IgG is not influenced by the various oral-related demographic factors evaluated.

Data presented in this study demonstrate for the first time that HPV-16– and HPV-18–specific antibodies induced by vaccination can be detected at the oral mucosa in 2 different oral specimen types. It is still uncertain whether the antibody levels measured in this study at the oral cavity are sufficient to provide long-term protection against acquisition of oral HPV-16 and HPV-18 infections. Future efficacy trials addressing the longevity of the immune responses in serum and at the oral cavity, as well as other aspects of B-cell immunity in relationship to HPV vaccine efficacy among men, are needed to inform vaccine protection against oral HPV infection and cancer in men.

## Supplementary Data

Supplementary materials are available at http://jid.oxfordjournals.org. Consisting of data provided by the author to benefit the reader, the posted materials are not copyedited and are the sole responsibility of the author, so questions or comments should be addressed to the author.

Supplementary Data
