# Supplementary material for: Quadrivalent Human Papillomavirus (HPV) Vaccine Induces HPV-Specific Antibodies in the Oral Cavity: Results From the Mid-Adult Male Vaccine Trial
Source: J Infect Dis. 2016 Aug 10;214(8):1276–83. doi: 10.1093/infdis/jiw359 (PMC5034962; doi:10.1093/infdis/jiw359)
Supplement: Supplementary Data [file supp_214_8_1276__index.html]

THE QUADRIVALENT HPV VACCINE INDUCES HPV-SPECIFIC ANTIBODIES AT THE ORAL CAVITY: RESULTS FROM THE MID-ADULT MALE VACCINE TRIAL-THE MAM TRIAL — Quadrivalent Human Papillomavirus (HPV) Vaccine Induces HPV-Specific Antibodies in the Oral Cavity: Results From the Mid-Adult Male Vaccine Trial — Supplementary Data 

# Quadrivalent Human Papillomavirus (HPV) Vaccine Induces HPV-Specific Antibodies in the Oral Cavity: Results From the Mid-Adult Male Vaccine Trial

## Supplementary Data

Supplementary Data

- Supplementary Data - Docx file
