# Supplementary material for: Quadrivalent Human Papillomavirus (HPV) Vaccine Induces HPV-Specific Antibodies in the Oral Cavity: Results From the Mid-Adult Male Vaccine Trial
Source: J Infect Dis. 2016 Aug 10;214(8):1276–83. doi: 10.1093/infdis/jiw359 (PMC5034962; doi:10.1093/infdis/jiw359)
Supplement: Supplementary Data [file supp_jiw359_jiw359supp.docx]

**Supplementary Content**

**Supplementary Figure 1.** IgG-normalized anti-HPV-16 and anti-HPV-18 antibodies in oral fluids and serum in vaccine recipients at Month 7.

**Supplementary Table 1.** HPV-16 and HPV-18 antibody levels (Month 7) among samples with detectable levels stratified by baseline subject characteristics.

**Supplementary Table 2.** IgG normalized-HPV-16 and HPV-18 Levels (Month 7) among samples with detectable levels stratified by baseline subject characteristics.

**Supplementary Figure 1.** **IgG-normalized anti-HPV-16 and anti-HPV-18 antibodies in oral fluids and serum in vaccine recipients at Month 7.**

Supplementary Figure 1. Following three doses of Gardasil, the participants’ samples (sponge, white bar; mouthwash, gray bar; and serum, black bar) were tested for anti-HPV-16 and HPV-18 antibodies and total IgG levels. Levels of antibodies determined by ELISA were normalized to total IgG levels in the respective samples, and the values are reported as antibody titer in EU/mL per mg of IgG. P values were calculated by Wilcoxon rank-sum test, and Bonferroni correction was used to set significance at p<0.0167.

**Supplementary Table 1: HPV-16 and HPV-18 antibody levels (Month 7) among samples with detectable levels stratified by baseline subject characteristics.**

| **Baseline Characteristics** | |  | **Sponge HPV-16** |  | **Sponge HPV-18** |  | **Oral Gargle HPV-16** |  | **Oral Gargle HPV-18** |  | **Serum HPV-16** | **Serum HPV-18** |
| --- | --- | --- | --- | --- | --- | --- | --- | --- | --- | --- | --- | --- |
|  |  | **N** | **GMT^b^ (95% CI)** | **N** | **GMT^b^ (95% CI)** | **N** | **GMT^b^ (95% CI)** | **N** | **GMT^b^ (95% CI)** | **N** | **GMT^b^ (95% CI)** | **GMT^b^ (95% CI)** |
| **Country** |  |  |  |  |  |  |  |  |  |  |  |  |
|  | **U.S.** | 59 | 2.49 (1.72, 3.58)**^a^** | 36 | 1.39 (0.95, 2.04)**^a^** | 62 | 0.17 (0.14, 0.20)**^a^** | 45 | 0.08 (0.07, 0.10)**^a^** | 51 | 2078.34 (1640.51, 2633.02) | 603.74 (466.19, 781.86) |
|  | **Mexico** | 74 | 6.60 (5.25, 8.28) | 55 | 3.26 (2.50, 4.24) | 75 | 0.33 (0.27, 0.41) | 61 | 0.15 (0.12, 0.18) | 75 | 2148.40 (1836.81, 2512.84) | 617.02 (512.43, 742.96) |
|  | **Total** | 133 | 4.28 (3.44, 5.33) | 91 | 2.33 (1.84, 2.93) | 137 | 0.24 (0.21, 0.28) | 106 | 0.12 (0.10, 0.13) | 126 | 2119.76 (1858.22, 2418.11) | 611.61 (526.41, 710.60) |
| **Age (yr)** |  |  |  |  |  |  |  |  |  |  |  |  |
|  | **26-30** | 30 | 4.87 (2.97, 7.99) | 18 | 2.45 (1.48, 4.05) | 37 | 0.19 (0.15, 0.26) | 27 | 0.10 (0.08, 0.13) | 31 | 2407.68 (1854.82, 3125.31) | 695.07 (540.97, 893.07) |
|  | **31-35** | 32 | 4.32 (2.79, 6.69) | 22 | 2.57 (1.65, 4.00) | 31 | 0.25 (0.18, 0.34) | 22 | 0.12 (0.08, 0.17) | 30 | 2010.03 (1534.67, 2632.63) | 537.01 (400.87, 719.39) |
|  | **36-40** | 52 | 4.10 (2.80, 6.02) | 38 | 2.25 (1.46, 3.48) | 51 | 0.28 (0.21, 0.36) | 43 | 0.13 (0.10, 0.17) | 47 | 1968.11 (1551.76, 2496.18) | 596.46 (443.67, 801.85) |
|  | **41-45** | 19 | 3.85 (2.27, 6.55) | 13 | 2.00 (1.23, 3.27) | 18 | 0.24 (0.14, 0.40) | 14 | 0.11 (0.07, 0.17) | 18 | 2257.75 (1593.52, 3198.84) | 650.72 (437.27, 968.36) |
| **BMI** |  |  |  |  |  |  |  |  |  |  |  |  |
|  | **Normal** | 57 | 4.65 (3.27, 6.61) | 38 | 2.78 (1.90, 4.07) | 61 | 0.26 (0.21, 0.33) | 47 | 0.13 (0.10, 0.16) | 57 | 2276.64 (1862.42, 2782.99) | 650.28 (525.38, 804.88) |
|  | **Overweight** | 48 | 4.58 (3.35, 6.27) | 36 | 2.29 (1.59, 3.29) | 48 | 0.26 (0.2, 0.34) | 40 | 0.11 (0.09, 0.14) | 46 | 2019.73 (1631.25, 2500.72) | 591.22 (455.78, 766.90) |
|  | **Obese** | 28 | 3.21 (1.81, 5.71) | 17 | 1.61 (0.93, 2.79) | 28 | 0.17 (0.13, 0.24) | 19 | 0.10 (0.07, 0.15) | 23 | 1956.27 (1388.69, 2755.84) | 562.26 (374.85, 843.36) |
| **Any alcohol** |  |  |  |  |  |  |  |  |  |  |  |  |
|  | **Yes** | 98 | 4.05 (3.16, 5.19) | 63 | 2.22 (1.74, 2.83) | 104 | 0.22 (0.19, 0.26) | 78 | 0.11 (0.09, 0.13) | 91 | 2070.4 (1773.48, 2417.04) | 574.99 (490.47, 674.07)**^a^** |
|  | **No** | 30 | 5.46 (3.18, 9.39) | 24 | 2.92 (1.59, 5.35) | 28 | 0.34 (0.23, 0.51) | 24 | 0.15 (0.10, 0.23) | 30 | 2394.93 (1798.54, 3189.10) | 768.71 (512.33, 1153.40) |
| **Total alcohol (drinks monthly)** |  |  |  |  |  |  |  |  |  |  |  |  |
|  | **0-10** | 37 | 4.44 (2.83, 6.95) | 25 | 2.40 (1.56, 3.70) | 37 | 0.24 (0.18, 0.32) | 30 | 0.11 (0.08, 0.15) | 36 | 2041.57 (1563.45, 2665.89) | 678.23 (543.65, 846.13) |
|  | **10+** | 59 | 3.73 (2.74, 5.07) | 37 | 2.05 (1.50, 2.79) | 65 | 0.21 (0.17, 0.26) | 47 | 0.10 (0.09, 0.13) | 53 | 2174.96 (1800.78, 2626.89) | 546.92 (444.55, 672.86) |
| **Ever gum disease** |  |  |  |  |  |  |  |  |  |  |  |  |
|  | **Yes** | 26 | 3.08 (1.97, 4.81) | 17 | 1.71 (0.98, 2.98) | 24 | 0.25 (0.18, 0.35) | 22 | 0.11 (0.08, 0.15) | 25 | 1918.99 (1492.55, 2467.26) | 635.00 (454.45, 887.30) |
|  | **No** | 102 | 4.74 (3.65, 6.14) | 70 | 2.59 (1.99, 3.39) | 108 | 0.24 (0.20, 0.29) | 80 | 0.12 (0.10, 0.14) | 96 | 2210.07 (1886.55, 2589.08) | 613.53 (513.81, 732.61) |
| **Tobacco use status** |  |  |  |  |  |  |  |  |  |  |  |  |
|  | **Current** | 34 | 6.76 (4.75, 9.63)^a^ | 27 | 3.02 (2.24, 4.06) | 33 | 0.37 (0.29, 0.48**)^a^** | 28 | 0.14 (0.11, 0.18) | 35 | 2292.45 (1860.14, 2825.23) | 647.23 (511.00, 819.78) |
|  | **Former** | 21 | 3.48 (1.96, 6.20) | 14 | 2.21 (1.16, 4.23) | 22 | 0.21 (0.13, 0.33) | 19 | 0.10 (0.07, 0.15) | 19 | 2154.58 (1431.85, 3242.13) | 614.96 (383.55, 985.99) |
|  | **Never** | 73 | 3.76 (2.73, 5.19) | 46 | 2.14 (1.46, 3.13) | 77 | 0.21 (0.17, 0.26) | 55 | 0.11 (0.09, 0.14) | 67 | 2071.81 (1707.44, 2513.94) | 603.94 (483.26, 754.75) |
| **Tooth loss** |  |  |  |  |  |  |  |  |  |  |  |  |
|  | **0 teeth** | 114 | 4.02 (3.16, 5.13) | 77 | 2.23 (1.73, 2.89) | 118 | 0.25 (0.21, 0.29) | 90 | 0.12 (0.10, 0.14) | 108 | 2131.03 (1848.05, 2457.33) | 615.80 (520.35, 728.76) |
|  | **1+ teeth** | 9 | 6.72 (3.07, 14.73) | 7 | 3.39 (1.48, 7.76) | 9 | 0.18 (0.09, 0.36) | 7 | 0.09 (0.05, 0.15) | 8 | 1766.09 (940.59, 3316.09) | 523.38 (326.18, 839.82) |
| **Gum bleeding** |  |  |  |  |  |  |  |  |  |  |  |  |
|  | **Yes** | 24 | 6.49 (3.71, 11.36) | 16 | 3.42 (1.56, 7.51) | 25 | 0.40 (0.26, 0.63)**^a^** | 22 | 0.17 (0.11, 0.27) | 24 | 2708.57 (1869.08, 3925.11) | 714.08 (463.66, 1099.74) |
|  | **No** | 104 | 3.95 (3.09, 5.06) | 71 | 2.21 (1.73, 2.81) | 107 | 0.22 (0.18, 0.25) | 80 | 0.10 (0.09, 0.12) | 97 | 2026.48 (1757.61, 2336.49) | 596.18 (505.78, 702.75) |
| **Teeth brushing (daily)** |  |  |  |  |  |  |  |  |  |  |  |  |
|  | **1** | 28 | 2.52 (1.35, 4.71)^a^ | 13 | 1.34 (0.63, 2.86)^a^ | 27 | 0.19 (0.13, 0.29) | 21 | 0.09 (0.06, 0.13) | 24 | 2588.77 (1802.33, 3718.38) | 793.14 (573.34, 1097.19) |
|  | **2** | 67 | 4.65 (3.39, 6.38) | 50 | 2.75 (1.95, 3.88) | 72 | 0.24 (0.19, 0.30) | 54 | 0.14 (0.11, 0.17) | 63 | 1910.56 (1560.29, 2339.46) | 553.40 (434.09, 705.51) |
|  | **2+** | 33 | 5.97 (4.54, 7.86) | 24 | 2.45 (1.81, 3.30) | 33 | 0.30 (0.24, 0.38) | 27 | 0.11 (0.08, 0.14) | 34 | 2333.54 (1958.53, 2780.34) | 635.50 (501.98, 804.53) |
| **Ever mouth warts** |  |  |  |  |  |  |  |  |  |  |  |  |
|  | **Yes** | 3 | 5.24 (0.12, 231.88) | 3 | 1.22 (0.12, 12.55) | 3 | 0.14 (0.01, 2.85) | 2 | 0.04 (0.00, 1.49) | 2 | 3991.33 (0.01, 1583037840.30)^a^ | 716.81 (0.21, 2480065.87)^a^ |
|  | **No** | 125 | 4.32 (3.44, 5.43) | 84 | 2.45 (1.92, 3.12) | 129 | 0.25 (0.21, 0.29) | 100 | 0.12 (0.10, 0.14) | 119 | 2124.26 (1857.61, 2429.17) | 616.37 (527.06, 720.80) |
| **Oral HPV-16 (at Day 1)** |  |  |  |  |  |  |  |  |  |  |  |  |
|  | **No** | 129 | 4.37 (3.49, 5.46) | 89 | 2.36 (1.87, 2.99) | 133 | 0.24 (0.21, 0.29) | 102 | 0.12 (0.10, 0.14) | 122 | 2126.53 (1864.88, 2424.90) | 614.51 (526.43, 717.33) |
|  | **Yes** | 4 | 2.25 (0.53, 9.59) | 2 | 1.13 (0.00, 1828978.39) | 4 | 0.15 (0.03, 0.79) | 4 | 0.07 (0.04, 0.12) | 4 | 1923.24 (259.08, 14276.71) | 529.44 (332.04, 844.20) |
| **Oral HPV-18 (at Day 1)** |  |  |  |  |  |  |  |  |  |  |  |  |
|  | **No** | 132 | 4.25 (3.41, 5.29) | 90 | 2.29 (1.81, 2.89) | 136 | 0.24 (0.20, 0.28) | 105 | 0.11 (0.10, 0.13) | 125 | 2101.08 (1842.05, 2396.53) | 602.26 (519.37, 698.39) |
| **Genital HPV-16 (at Day 1)** |  |  |  |  |  |  |  |  |  |  |  |  |
|  | **No** | 118 | 4.41 (3.47, 5.59) | 82 | 2.46 (1.92, 3.15) | 122 | 0.24 (0.20, 0.28) | 93 | 0.12 (0.10, 0.14) | 113 | 2070.93 (1808.64, 2371.26) | 615.08 (522.92, 723.48) |
|  | **Yes** | 15 | 3.40 (1.91, 6.05) | 9 | 1.39 (0.70, 2.75) | 15 | 0.25 (0.16, 0.39) | 13 | 0.09 (0.07, 0.12) | 13 | 2595.77 (1494.84, 4507.53) | 582.24 (386.55, 876.99) |
| **Genital HPV-18 (at Day 1)** |  |  |  |  |  |  |  |  |  |  |  |  |
|  | **No** | 133 | 4.28 (3.44, 5.33) | 91 | 2.33 (1.84, 2.93) | 137 | 0.24 (0.21, 0.28) | 106 | 0.12 (0.10, 0.13) | 126 | 2119.76 (1858.22, 2418.11) | 611.61 (526.41, 710.60) |

| **Supplementary Table 1: HPV-16 and HPV-18 antibody levels (Month 7) among samples with detectable levels stratified by baseline subject characteristics. (continued)** | | | | | | | | | | | | | | |
| --- | --- | --- | --- | --- | --- | --- | --- | --- | --- | --- | --- | --- | --- | --- |
| **Baseline Characteristics** | |  | **Sponge HPV-16** |  | **Sponge HPV-18** | |  | **Oral Gargle HPV-16** |  | **Oral Gargle HPV-18** | |  | **Serum HPV-16** | **Serum HPV-18** |
|  |  | **N** | **GMT^b^ (95% CI)** | **N** | **GMT^b^ (95% CI)** | | **N** | **GMT^b^ (95% CI)** | **N** | **GMT^b^ (95% CI)** | | **N** | **GMT^b^ (95% CI)** | **GMT^b^ (95% CI)** |
| **HPV-16 Seropositive (at Day 1)** |  |  |  |  | |  |  |  | |  |  |  |  |  |
|  | **No** | 126 | 4.18 (3.33, 5.24) | 84 | | 2.39 (1.87, 3.04) | 137 | 0.24 (0.21, 0.28) | | 106 | 0.12 (0.10, 0.13) | 102 | 2071.95 (1795.56, 2390.89) | 616.17 (522.63, 726.45) |
|  | **Yes** | 7 | 6.67 (2.45, 18.15) | 7 | | 1.71 (0.63, 4.65) |  |  | |  |  | 24 | 2335.58 (1646.18, 3313.7) | 592.60 (401.72, 874.18) |
| **HPV-18 Seropositive (at Day 1)** |  |  |  |  | |  |  |  | |  |  |  |  |  |
|  | **No** | 122 | 4.18 (3.32, 5.26) | 81 | | 2.32 (1.81, 2.97) | 131 | 0.24 (0.21, 0.28) | | 101 | 0.12 (0.10, 0.14) | 100 | 2098.22 (1805.46, 2438.45) | 598.07 (503.97, 709.74) |
|  | **Yes** | 11 | 5.63 (2.44, 12.98) | 10 | | 2.41 (1.07, 5.43) | 6 | 0.26 (0.1, 0.70) | | 5 | 0.12 (0.07, 0.21) | 26 | 2204.70 (1649.61, 2946.58) | 666.60 (479.83, 926.06) |

^a^  Indicates p<0.05, Wilcoxon/Kruskal-Wallis test.

^b^ GMT represents the geometric mean concentrations amongst positive values (above cutoff).

**Supplementary Table 2. IgG normalized-HPV-16 and HPV-18 Levels (Month 7) among samples with detectable levels stratified by baseline subject characteristics.**

| **Baseline Characteristic** | |  | **Sponge HPV-16** |  | **Sponge HPV-18** |  | **Oral Gargle HPV-16** |  | **Oral Gargle HPV-18** |  | **Serum HPV-16** | **Serum HPV-18** |
| --- | --- | --- | --- | --- | --- | --- | --- | --- | --- | --- | --- | --- |
|  |  | **N** | **GMT^a^ (EU/mg) with 95% CI** | **N** | **GMT^a^ (EU/mg) with 95% CI** | **N** | **GMT^a^ (EU/mg) with 95% CI** | **N** | **GMT^a^ (EU/mg) with 95% CI** | **N** | **GMT^a^ (EU/mg) with 95% CI** | **GMT^a^ (EU/mg) with 95% CI** |
| **Country** |  |  |  |  |  |  |  |  |  |  |  |  |
|  | **U.S.** | 59 | 226.97 (185.04, 278.40) | 36 | 94.78 (70.74, 126.98) | 62 | 192.17 (159.85, 231.01) | 45 | 83.91 (68.06, 103.46) | 51 | 251.92 (195.41, 324.77) | 73.18 (55.72, 96.12) |
|  | **Mexico** | 74 | 221.44 (188.81, 259.71) | 55 | 101.23 (84.72, 120.97) | 75 | 165.55 (139.34, 196.69) | 61 | 66.93 (55.94, 80.07) | 75 | 272.68 (233.09, 319.01) | 78.31 (64.97, 94.41) |
|  | **Total** | 133 | 223.87 (197.55, 253.71) | 91 | 98.63 (84.49, 115.13) | 137 | 177.11 (156.31, 200.67) | 106 | 73.67 (64.32, 84.37) | 126 | 264.08 (230.29, 302.83) | 76.19 (65.29, 88.92) |
| **Age (yr)** |  |  |  |  |  |  |  |  |  |  |  |  |
|  | **26-30** | 30 | 232.75 (186.81, 289.98) | 18 | 96.94 (69.92, 134.39) | 37 | 166.46 (134.74, 205.66) | 27 | 70.07 (53.88, 91.13) | 31 | 298.73 (227.44, 392.35) | 86.24 (66.01, 112.67) |
|  | **31-35** | 32 | 216.97 (168.54, 279.33) | 22 | 89.73 (62.00, 129.87) | 31 | 189.25 (152.15, 235.41) | 22 | 71.05 (50.50, 99.96) | 30 | 242.79 (181.39, 324.96) | 64.86 (47.75, 88.11) |
|  | **36-40** | 52 | 206.07 (163.58, 259.60) | 38 | 97.65 (75.28, 126.67) | 51 | 170.97 (133.96, 218.22) | 43 | 75.37 (59.90, 94.84) | 47 | 249.19 (195.58, 317.49) | 75.52 (55.76, 102.27) |
|  | **41-45** | 19 | 278.46 (199.89, 387.90) | 13 | 122.05 (86.98, 171.25) | 18 | 198.28 (132.81, 296.03) | 14 | 80.05 (56.84, 112.75) | 18 | 285.90 (199.61, 409.48) | 82.40 (56.22, 120.77) |
| **BMI** |  |  |  |  |  |  |  |  |  |  |  |  |
|  | **Normal** | 57 | 247.26 (200.27, 305.27) | 38 | 115.95 (90.55, 148.47) | 61 | 200.33 (164.32, 244.23) | 47 | 83.91 (66.54, 105.82) | 57 | 288.78 (234.13, 356.18) | 82.48 (66.45, 102.38) |
|  | **Overweight** | 48 | 225.14 (190.29, 266.38) | 36 | 90.24 (71.13, 114.50) | 48 | 174.28 (144.60, 210.06) | 40 | 63.92 (52.30, 78.12) | 46 | 258.63 (209.60, 319.12) | 75.71 (58.20, 98.49) |
|  | **Obese** | 28 | 181.11 (132.81, 246.99) | 17 | 82.92 (55.64, 123.57) | 28 | 139.19 (102.88, 188.33) | 19 | 71.98 (53.94, 96.06) | 23 | 220.60 (151.48, 321.28) | 63.40 (41.00, 98.05) |
| **Any alcohol** |  |  |  |  |  |  |  |  |  |  |  |  |
|  | **Yes** | 98 | 222.68 (192.41, 257.72) | 63 | 94.47 (78.85, 113.19) | 104 | 171.36 (148.42, 197.85) | 78 | 68.88 (59.02, 80.39) | 91 | 259.42 (220.13, 305.73) | 72.05 (60.94, 85.17) |
|  | **No** | 30 | 241.05 (184.18, 315.47) | 24 | 113.84 (80.08, 161.84) | 28 | 217.03 (165.30, 284.93) | 24 | 97.62 (71.83, 132.68) | 30 | 291.99 (220.33, 386.95) | 93.72 (62.74, 140.00) |
| **Total alcohol (drinks monthly)** |  |  |  |  |  |  |  |  |  |  |  |  |
|  | **0-10** | 37 | 233.26 (182.23, 298.57) | 25 | 108.82 (83.51, 141.80) | 37 | 174.48 (135.77, 224.22) | 30 | 70.12 (56.02, 87.78) | 36 | 265.92 (198.87, 355.58) | 88.34 (70.11, 111.31) |
|  | **10+** | 59 | 224.06 (186.90, 268.61) | 37 | 86.49 (66.96, 111.72) | 65 | 175.58 (147.83, 208.53) | 47 | 69.22 (55.74, 85.97) | 53 | 265.97 (218.66, 323.52) | 66.88 (53.79, 83.15) |
| **Ever gum disease** |  |  |  |  |  |  |  |  |  |  |  |  |
|  | **Yes** | 26 | 193.92 (148.96, 252.46) | 17 | 90.43 (58.25, 140.38) | 24 | 157.15 (124.14, 198.93) | 22 | 60.05 (44.13, 81.72) | 25 | 236.44 (180.25, 310.14) | 78.24 (54.96, 111.37) |
|  | **No** | 102 | 236.11 (204.18, 273.03) | 70 | 101.79 (85.56, 121.09) | 108 | 185.73 (160.42, 215.02) | 80 | 79.42 (67.92, 92.87) | 96 | 275.77 (234.15, 324.79) | 76.56 (63.89, 91.73) |
| **Tobacco use status** |  |  |  |  |  |  |  |  |  |  |  |  |
|  | **Current** | 34 | 242.47 (198.55, 296.10) | 27 | 85.41 (68.27, 106.84) | 33 | 201.60 (163.57, 248.47) | 28 | 69.61 (54.73, 88.53) | 35 | 297.81 (244.09, 363.35) | 84.08 (67.33, 104.99) |
|  | **Former** | 21 | 224.00 (161.48, 310.73) | 14 | 115.29 (75.30, 176.51) | 22 | 170.29 (115.69, 250.66) | 19 | 78.15 (57.71, 105.82) | 19 | 267.95 (174.30, 411.92) | 76.48 (46.13, 126.79) |
|  | **Never** | 73 | 220.74 (183.42, 265.64) | 46 | 103.98 (80.92, 133.62) | 77 | 174.48 (147.06, 207.03) | 55 | 76.37 (61.86, 94.29) | 67 | 252.18 (205.53, 309.43) | 73.51 (58.40, 92.54) |
| **Tooth loss** |  |  |  |  |  |  |  |  |  |  |  |  |
|  | **0 teeth** | 114 | 226.23 (197.81, 258.73) | 77 | 97.70 (81.85, 116.61) | 118 | 183.50 (160.75, 209.47) | 90 | 76.09 (65.36, 88.58) | 108 | 266.03 (229.35, 308.56) | 76.87 (64.69, 91.35) |
|  | **1+ teeth** | 9 | 200.12 (105.58, 379.32) | 7 | 104.78 (67.46, 162.74) | 9 | 129.86 (64.80, 260.25) | 7 | 56.89 (35.27, 91.75) | 8 | 219.65 (117.26, 411.44) | 65.09 (40.65, 104.24) |
| **Gum bleeding** |  |  |  |  |  |  |  |  |  |  |  |  |
|  | **Yes** | 24 | 247.83 (171.60, 357.92) | 16 | 112.82 (66.77, 190.64) | 25 | 181.75 (122.69, 269.23) | 22 | 72.09 (48.80, 106.49) | 24 | 339.13 (232.07, 495.58) | 89.41 (57.53, 138.95) |
|  | **No** | 104 | 222.27 (194.35, 254.21) | 71 | 96.67 (82.04, 113.92) | 107 | 179.80 (157.82, 204.84) | 80 | 75.53 (65.24, 87.44) | 97 | 251.83 (216.95, 292.31) | 74.09 (62.57, 87.72) |
| **Teeth brushing (daily)** |  |  |  |  |  |  |  |  |  |  |  |  |
|  | **1** | 28 | 231.32 (168.47, 317.61) | 13 | 121.85 (75.68, 196.18) | 27 | 179.82 (131.88, 245.19) | 21 | 73.40 (55.02, 97.93) | 24 | 306.81 (207.31, 454.07) | 94.00 (66.05, 133.77) |
|  | **2** | 67 | 218.60 (180.65, 264.51) | 50 | 98.01 (77.95, 123.24) | 72 | 175.95 (145.64, 212.55) | 54 | 82.07 (66.62, 101.10) | 63 | 244.12 (197.79, 301.30) | 70.71 (55.06, 90.81) |
|  | **2+** | 33 | 240.59 (201.18, 287.72) | 24 | 91.87 (71.03, 118.82) | 33 | 190.04 (157.86, 228.77) | 27 | 62.97 (49.09, 80.77) | 34 | 286.30 (239.76, 341.88) | 77.97 (61.84, 98.30) |
| **Ever mouth warts** |  |  |  |  |  |  |  |  |  |  |  |  |
|  | **Yes** | 3 | 300.23 (12.83, 7024.00) | 3 | 70.04 (16.88, 290.56) | 3 | 188.02 (5.03, 7024.21) | 2 | 64.71 (0.12, 34366.89) | 2 | 416.78 (0.00, 96707191.83) | 74.85 (0.04, 151506.30) |
|  | **No** | 125 | 225.34 (198.57, 255.71) | 84 | 100.71 (85.47, 118.68) | 129 | 179.99 (158.74, 204.08) | 100 | 74.99 (65.13, 86.34) | 119 | 265.15 (230.44, 305.09) | 76.94 (65.51, 90.35) |
|  |  |  |  |  |  |  |  |  |  |  |  |  |
|  | | | | | | | | | | | | |
|  | | | | | | | | | | | | |
| **Supplementary Table 2: IgG normalized-HPV-16 and HPV-18 Levels (Month 7) among samples with detectable levels stratified by baseline subject characteristics. (continued)** | | | | | | | | | | | | |
| **Baseline Characteristic** | |  | **Sponge HPV-16** |  | **Sponge HPV-18** |  | **Oral Gargle HPV-16** |  | **Oral Gargle HPV-18** |  | **Serum HPV-16** | **Serum HPV-18** |
|  |  | **N** | **GMT^a^ (EU/mg) with 95% CI** | **N** | **GMT^a^ (EU/mg) with 95% CI** | **N** | **GMT^a^ (EU/mg) with 95% CI** | **N** | **GMT^a^ (EU/mg) with 95% CI** | **N** | **GMT^a^ (EU/mg) with 95% CI** | **GMT^a^ (EU/mg) with 95% CI** |
| **Oral HPV-16 (at Day 1)** |  |  |  |  |  |  |  |  |  |  |  |  |
|  | **No** | 129 | 224.51 (198.11, 254.44) | 89 | 99.13 (84.66, 116.07) | 133 | 178.86 (158.20, 202.21) | 102 | 74.34 (64.64, 85.49) | 122 | 264.81 (231.01, 303.55) | 76.52 (65.24, 89.75) |
|  | **Yes** | 4 | 204.17 (31.40, 1327.53) | 2 | 78.67 (1.08, 5750.70) | 4 | 127.72 (12.69, 1285.55) | 4 | 58.46 (26.59, 128.55) | 4 | 242.87 (30.30, 1946.89) | 66.86 (48.75, 91.70) |
| **Oral HPV-18 (at Day 1)** |  |  |  |  |  |  |  |  |  |  |  |  |
|  | **No** | 132 | 222.18 (196.04, 251.79) | 90 | 96.90 (83.20, 112.85) | 136 | 176.05 (155.32, 199.55) | 105 | 72.71 (63.56, 83.17) | 125 | 261.63 (228.18, 299.98) | 75.00 (64.39, 87.35) |
| **Genital HPV-16 (at Day 1)** |  |  |  |  |  |  |  |  |  |  |  |  |
|  | **No** | 118 | 228.92 (200.46, 261.42) | 82 | 102.86 (87.71, 120.61) | 122 | 176.55 (154.29, 202.03) | 93 | 76.03 (65.72, 87.96) | 113 | 258.81 (225.40, 297.18) | 76.87 (65.17, 90.66) |
|  | **Yes** | 15 | 187.86 (124.68, 283.07) | 9 | 67.29 (35.20, 128.63) | 15 | 181.66 (126.81, 260.23) | 13 | 58.78 (39.38, 87.72) | 13 | 314.63 (166.32, 595.20) | 70.57 (43.04, 115.72) |
| **Genital HPV-18 (at Day 1)** |  |  |  |  |  |  |  |  |  |  |  |  |
|  | **No** | 133 | 223.87 (197.55, 253.71) | 91 | 98.63 (84.49, 115.13) | 137 | 177.11 (156.31, 200.67) | 106 | 73.67 (64.32, 84.37) | 126 | 264.08 (230.29, 302.83) | 76.19 (65.29, 88.92) |
| **HPV-16 Seropositive (at Day 1)** |  |  |  |  |  |  |  |  |  |  |  |  |
|  | **No** | 126 | 225.83 (199.05, 256.20) | 84 | 104.56 (89.40, 122.30) | 137 | 177.11 (156.31, 200.67) | 106 | 73.67 (64.32, 84.37) | 102 | 260.59 (225.03, 301.77) | 77.50 (65.54, 91.63) |
|  | **Yes** | 7 | 191.45 (75.98, 482.43) | 7 | 48.91 (26.36, 90.74) |  |  |  |  | 24 | 279.42 (190.46, 409.92) | 70.90 (46.77, 107.46) |
| **HPV-18 Seropositive (at Day 1)** |  |  |  |  |  |  |  |  |  |  |  |  |
|  | **No** | 122 | 226.63 (198.95, 258.16) | 81 | 102.84 (87.55, 120.79) | 131 | 182.07 (160.62, 206.37) | 101 | 74.92 (65.24, 86.05) | 100 | 265.06 (226.17, 310.63) | 75.55 (63.17, 90.36) |
|  | **Yes** | 11 | 195.48 (115.69, 330.30) | 10 | 70.30 (39.13, 126.31) | 6 | 96.87 (39.15, 239.67) | 5 | 52.37 (20.40, 134.46) | 26 | 260.36 (196.64, 344.72) | 78.72 (57.39, 107.97) |

^a^ GMT represents the geometric mean concentrations amongst positive values (above cutoff), and GMT values are normalized to total IgG levels in the corresponding sample type.
